# Incidence and time-varying predictors of HIV and sexually transmitted infections among male sex workers in Mexico City

**DOI:** 10.1186/s40249-020-00792-2

**Published:** 2021-01-19

**Authors:** Karla Y. Ganley, Marta Wilson-Barthes, Andrew R. Zullo, Sandra G. Sosa-Rubí, Carlos J. Conde-Glez, Santa García-Cisneros, Mark N. Lurie, Brandon D. L. Marshall, Don Operario, Kenneth H. Mayer, Omar Galárraga

**Affiliations:** 1 Department of Narrative Medicine, Columbia University, New York, NY USA; 2 Department of Epidemiology, School of Public Health, Brown University, Providence, RI USA; 3 Department of Health Services, Policy, and Practice, School of Public Health, Brown University, 121 South Main Street, Box G-121S-2, Providence, RI 02912 USA; 4 National Institute of Public Health (INSP), Cuernavaca, Morelos Mexico; 5 Department of Behavioral and Social Science, School of Public Health, Brown University, Providence, RI USA; 6 Fenway Health and Harvard University, Boston, MA USA

**Keywords:** Male sex worker, Men who have sex with men, HIV, Sexually transmitted infection, Transmission, Risk factor, Mexico

## Abstract

**Background:**

Male sex workers are at high-risk for acquisition of sexually transmitted infections (STIs), including human immunodeficiency virus (HIV). We quantified incidence rates of STIs and identified their time-varying predictors among male sex workers in Mexico City.

**Methods:**

From January 2012 to May 2014, male sex workers recruited from the largest HIV clinic and community sites in Mexico City were tested for chlamydia, gonorrhea, syphilis, hepatitis, and HIV at baseline, 6-months, and 12-months. Incidence rates with 95% bootstrapped confidence limits were calculated. We examined potential time-varying predictors using generalized estimating equations for a population averaged model.

**Results:**

Among 227 male sex workers, median age was 24 and baseline HIV prevalence was 32%. Incidence rates (per 100 person-years) were as follows: HIV [5.23; 95% confidence interval (*CI*): 2.15–10.31], chlamydia (5.15; 95% *CI*: 2.58–9.34), gonorrhea (3.93; 95% *CI*: 1.88–7.83), syphilis (13.04; 95% *CI*: 8.24–19.94), hepatitis B (2.11; 95% *CI*: 0.53–4.89), hepatitis C (0.95; 95% *CI*: 0.00–3.16), any STI except HIV (30.99; 95% *CI*: 21.73–40.26), and any STI including HIV (50.08; 95% *CI*: 37.60–62.55). In the multivariable-adjusted model, incident STI (excluding HIV) were lower among those who reported consistently using condoms during anal and vaginal intercourse (odds ratio = 0.03, 95% *CI*: 0.00–0.68) compared to those who reported inconsistently using condoms during anal and vaginal intercourse.

**Conclusions:**

Incidence of STIs is high among male sex workers in Mexico City. Consistent condom use is an important protective factor for STIs, and should be an important component of interventions to prevent incident infections.

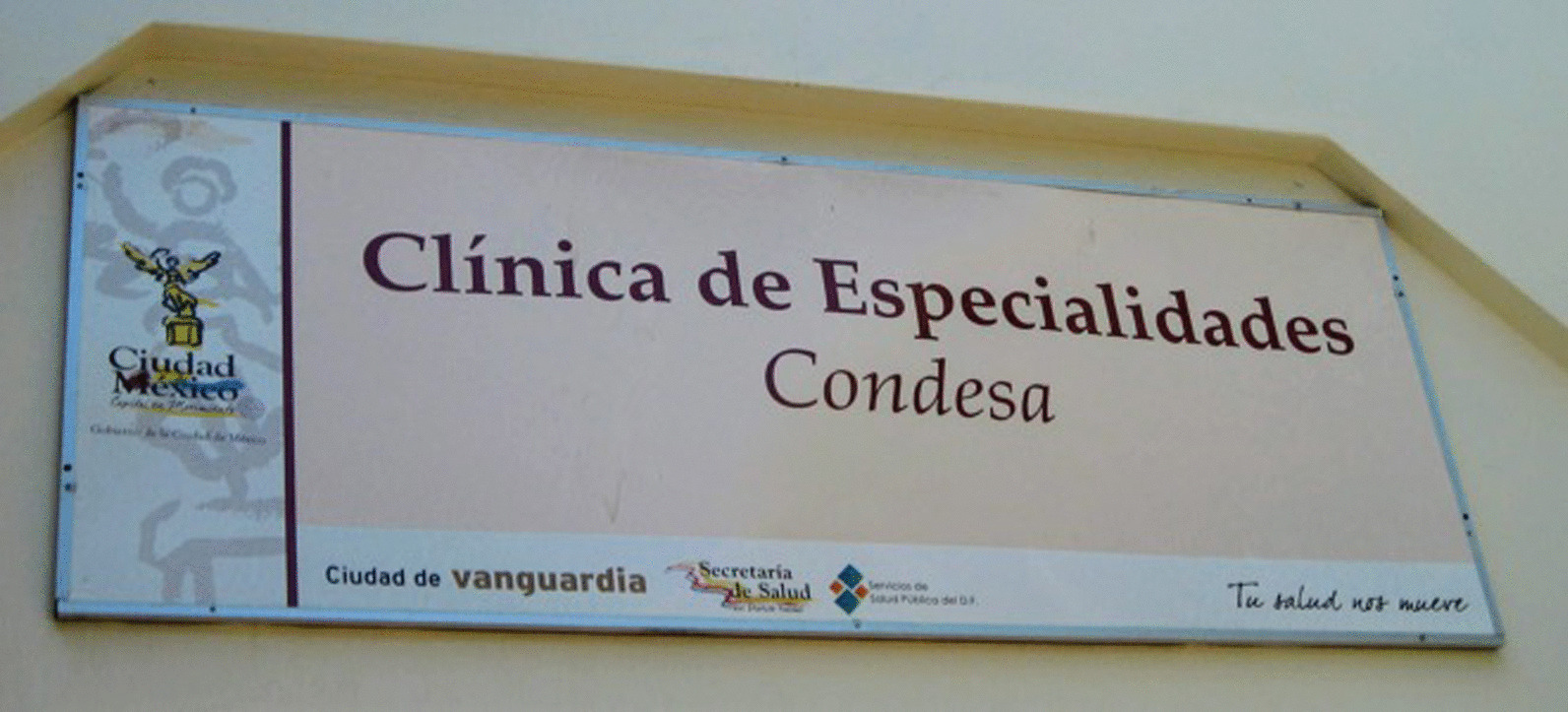

## Background

The prevalence of human immunodeficiency virus (HIV) in Mexico is 0.3% in the general adult population, 16.9% among men who have sex with men (MSM), and 18.2% among male sex workers (MSWs) [1, 2]. Studies at the global level have shown that, despite a decline in HIV infection in recent years among the general adult population, HIV acquisition among MSWs has increased [3]. In part, this is a result of higher transmissibility of HIV during anal intercourse, as well as other risk factors among MSWs, including multiple sexual partnerships, membership in dense sexual networks, and limited access to healthcare services due to stigma [4, 5]. Since these factors are predictors of acquisition of both HIV and other sexually transmitted infections (STIs), it is likely that MSWs in Mexico City are at an increased risk of all STIs, not just HIV [6, 7]. However, scant information is available about the incidence of STIs among MSWs in Mexico City. This is partly due to underreporting of STIs in Latin American countries, including Mexico, because national STI programs lack consistency about which STIs are reportable by law, and definitions of these STIs—clinical versus syndromic versus laboratory based [8]. Information is also limited because MSWs are a highly vulnerable and stigmatized population, leading few MSWs to openly disclose their occupation as sex workers [9, 10]. It is also important to note that “sex worker” may be a vague category and, as such, the precise definition for MSWs can vary according to study. For the purposes of this study, MSWs are defined as cisgender men, ages 18–40, who either self-identified as MSWs or who did not self-identify as MSWs, but who declared that they were a man who had sex with a male partner in exchange for money in the past six months and who had at least 10 male sexual partners within the last month. See the “Methods” section for further detail.

Previous studies have quantified STI prevalence among MSWs; however, no studies have examined STI incidence rates among MSWs in Mexico City, and this information is essential for health interventions. Furthermore, no studies have identified predictors of STI acquisition in MSWs in Mexico City or determined how predictors of STIs vary with time [11, 12]. MSWs are at high risk for HIV/STI acquisition due to their behavioral transactions with paying and non-paying male and female partners; and can be at risk for transmitting HIV/STIs to other populations. For example, a modeling study (using data from the same study population described below), estimated high rates of HIV transmission from MSWs to their clients and non-paying partners (approximately 8% per year) [13]. Identifying predictors of STI incidence is thus essential for developing and targeting interventions to reduce acquisition and onward transmission within MSWs/MSM populations as well as transmission to female populations [14–18]. Therefore, our study aims to determine STI/HIV incidence rates, and identify time-varying predictors of incident STI infection among a sample of MSWs in Mexico City.

## Methods

### Study setting and population

A detailed description of the study population and methods is available elsewhere; a brief overview is provided here [1, 19]. This study is a secondary analysis of a randomized controlled trial (RCT) that evaluated the impact of conditional economic incentives on staying free of new curable STIs among MSWs in Mexico City. We conducted this secondary analysis because there is a dearth of information about prevalence and incidence of STIs among MSWs. The RCT was not powered to analyze the effects on incident STI/HIV by study arm. Thus, we report here the incidence of STI/HIV and its determinants for the entire sample. This study took place from January 2012 to May 2014. Participants were recruited by trained research staff from community sites where MSWs were known to congregate in Mexico City, as determined in previous studies [19, 20]. Participants were also recruited through referral to the research team from within the *Condesa* HIV Testing Clinic. Participants were tested and treated for STIs, as indicated, at *Clínica Condesa*. Treatment was provided free of charge, including antiretroviral treatment for those identified as HIV-positive. All participants provided informed consent. All procedures were approved by Institutional Review Boards at Brown University in Providence, United States of America, and the National Institute of Public Health in Cuernavaca, Mexico.

The study population consisted of a convenience sample of 227 cisgender men, ages 18–40, who attended a clinic appointment at *Clinic Condesa*, and who either self-identified as MSWs (*n* = 152) or who did not self-identify as a MSWs, but who declared that they were a man who had sex with a male partner in exchange for money in the past six months and who had at least 10 male sexual partners within the last month (*n* = 75). Note that these criteria for inclusion in the study allow for participants that have sex with women in addition to men (hence, some study variables include vaginal intercourse), but who nevertheless meet the study’s definition of male sex worker. These criteria were determined based a previous study involving observations and in-depth interviews with sex workers in Mexico City [20]. Transgender women were excluded from the present study because *Clínica Condesa* has a separate program for them.

At the baseline visit, participants filled out a survey with questions regarding sociodemographic characteristics and health behaviors. At baseline (0 months) and follow-up visits one (6 months) and two (12 months), participants filled out the survey again and were tested and treated (as indicated) for syphilis, chlamydia, gonorrhea, and HIV.

### Data collection and measures

Data collection was done in partnership with the Mexican National Institute of Public Health (INSP) and the Consortium for HIV/AIDS and Tuberculosis Research (CISIDAT). Participants were administered the survey using laptop computers with audio computer assisted interviewing (A-CASI) questionnaires. All variables were assessed for missingness, range, and distribution. Blood and urine samples were obtained from the participants using bio-safety protocols. Samples were analyzed by trained laboratory personnel.

The main outcome of interest was new, confirmed cases of STIs and HIV. Urine specimens were tested for gonorrhea and chlamydia at the INSP Laboratory (PCR Cobas-Amplicor; Roche, Basel, Switzerland); and blood specimens served to measure the presence of HIV, hepatitis B, hepatitis C and syphilis antibodies at the Condesa Clinic Laboratory [Abbott HIV-1 and HIV-2, Ag/Ab Combo, anti-HBc, anti-HCV and syphilis TP quimioluminiscence immunoassay (Abbott Laboratories, North Chicago, IL, USA)] running in Architect i2000 (Abbott, North Chicago, IL, USA); HIV-positive samples were confirmed with HIV-1 and HIV-2 CombFirm (Orgenics, Alere, Israel). Anti-HBc+ was tested with determine HBsAg and syphilis TP+ (Abbott) with tittered venereal disease research laboratory (VDRL) test (titre ≥ 1:8 was used as the cut-off for active infection). At the baseline survey, two subgroups were defined for the markers of syphilis and hepatitis B: antibody positivity was regarded as a lifetime marker of past or present infection, whereas treponemic antibody positivity together with VDRL demonstrated active syphilis, and anti-HBc plus HBsAg positivity indicated current hepatitis B virus infection.

Based on findings from prior literature and known associations between specific sexual risk behaviors and incident STIs, we created a conceptual framework of likely predictors, the majority of which were time-varying, and included: age, education, drug use, condom use, and frequency and types of sexual [7, 21]. The demographic variable age was continuous and the other demographic variable, highest educational attainment, was categorical. Four separate variables were included to describe sexual activity: had vaginal, anal, or oral sex with clients last week; had vaginal, anal, or oral sex with non-paying partners in the past week; had insertive anal sex with any of three most recent clients; and had receptive anal sex with any of three most recent clients. The first two variables describing sexual activity were continuous variables and the latter two were binary variables. Consistent condom use during sex in the past month and drug use with any of three most recent clients were similarly coded as binary variables. See Additional file 1 for further details on each of these variables.

Lastly, since this study is a secondary analysis of a RCT that evaluated the impact of conditional economic incentives on staying free of new curable STIs, a variable for randomization to the four study arms of the original RCT was included in our model. This controls for the effect of incentives and conditionalities. Detailed descriptions of the main covariates and outcomes variables in this study are provided in Additional file 1. Income and wealth were not included in the model because nonresponse was high for these variables.

### Statistical analyses

Incidence rates were estimated using the person-time method (i.e., by dividing the total number of new HIV/STI infections observed during the study period by the total number of person years at risk). We calculated 95% confidence limits using a bias-corrected and accelerated bootstrap method with 1000 replicates [22, 23]. We chose this method because it yields appropriate confidence intervals even with relatively small sample sizes. Participants lost to follow-up stopped accruing person years at their last known study visit. To calculate person-time for seroconversions that happened during follow-up intervals, we took the midpoint of the follow-up interval as an estimate of the time at which seroconversion occurred. During the course of the study, 43 participants were lost to follow up at the 6-month visit and an additional 25 participants were lost to follow up at the 12-month visit. A detailed analysis of loss to follow up for this cohort was conducted in a previous study [24]. Participants with prevalent HIV infection at baseline were included in the analyses for incident STIs, but were excluded for analyses estimating HIV incidence. Participants with prevalent STIs at baseline were excluded for the STIs for which they tested positive, but were still included for calculations of incident STIs for which they tested negative at baseline. Since HIV is an incurable STI, someone diagnosed with HIV at six months would test positive again for HIV at 12 months. Thus, once diagnosed with HIV, individuals were excluded for analyses estimating HIV incidence but were included in the analyses for other incident STIs.

We estimated marginal models using generalized estimating equations (GEE) with a logit link and binomial variance to examine unadjusted and multivariable-adjusted time-varying predictors of incident STIs [25]. The GEE model provides marginal estimates, for which the estimate is averaged over all values of the covariates, which could be correlated. All models used an unstructured correlation structure. In the main analysis, we used a composite STIs outcome, and then we excluded HIV prevalent cases in a secondary analysis to examine combined incident STIs/HIV. The results of our present GEE model are conditional on returning to the Clinic for follow-up. We used quasi-likelihood independence model criterion (QIC) to select the best working correlation structure and the best subset of covariates as diagnostic measures of model fit [26]. Data were analyzed using STATA 13.1 (StataCorp LP, College Station, Texas, USA) and SAS 9.4 (SAS Institute Inc., Cary, NC, USA).

## Results

### Social and behavioral characteristics at baseline

Sociodemographic and behavioral characteristics at baseline are shown in Table 1. The median age among MSWs was 24 years, and HIV prevalence was 32%. The highest level of schooling for 34% of respondents was high school, while 18% of respondents had attended college or post-graduate school. The majority of MSWs (75%) were unmarried, yet 43% of MSWs reported having a stable romantic (i.e., non-paying) partner. MSWs had a median of three sexual partners in the last week. About a fifth of MSWs (22%) reported being intoxicated while having sex with any of their three most recent clients, and 22% reported taking drugs before sex with any of their three most recent clients. The minority of MSWs (29%) reported consistent condom use during sex in the past month. Additionally, 41% of MSWs had insertive anal sex with any of their three most recent clients, 51% had receptive anal sex, and 26% had both insertive and receptive anal sex with any of their three most recent clients.

**Table 1:** Baseline characteristics of male sex workers (*n* = 227) in Mexico City

Characteristic	*n* (%)
Demographics	
Age, years	24 (20–27), Median (IQR)
Highest educational attainment	
Primary or secondary school	92 (40.53)
High school	77 (33.92)
College or post-graduate	41 (18.06)
Marital status	
Single	171 (75.33)
Married/free union	42 (18.50)
Divorced/separated	2 (0.88)
Stable non-paying partner	97 (42.73)
*S*exual behaviors	
Number of male clients in the past week	4.55 (4.98), % Mean (SD)
Number of female clients in the past week	2.53 (6.03) % Mean (SD)
Number of people individual had vaginal or anal or oral sex with last week	3 (1–6), Median (IQR)
Intoxicated while having sex with any of three most recent clients	49 (21.59)
Used drugs before having sex with any of three most recent clients	49 (21.59)
Consistently used condoms during sex in past month	65 (28.63)
Had insertive anal sex with any of three most recent clients	94 (41.41)
Had receptive anal sex with any of three most recent clients	116 (51.10)
Had receptive and insertive anal sex with any of three most recent clients	58 (25.55)
STI prevalence confirmed via laboratory testing	
Positive STI test result	
HIV	73 (32.16)
Chlamydia	23 (10.13)
Gonorrhea	4 (1.76)
Active syphilis	42 (18.50)
Hepatitis B	20 (8.81)
Hepatitis C	2 (0.88)
Any STI (except HIV)	76 (33.48)
Any STI (including HIV)	116 (51.10)
Symptoms of STIs based on self report^a^	
Presence of burning during urination or penile discharge	15 (6.61)
Presence of sores, ulcers, or rash in or around the penis or rectum	21 (9.25)

Table shows number of cases and percentage in parentheses unless otherwise noted

*n* total respondents, *IQR* interquartile range, *SD* standard deviation, *STIs* sexually transmitted infections

^a^ Self-reported symptoms of STIs represent those reported on the 12-month survey

### Incidence rates for HIV and other STIs

The total amount of follow-up time for the study cohort was 215.63 person-years (PY), and the average follow-up per participant was 346.72 days. The highest incidence rates were for active syphilis (13.04 per 100 PY; 95%* CI*: 8.24–19.94) among the entire sample, and HIV (5.23 per 100 PY; 95%* CI*: 2.15–10.31) among the HIV-susceptible sample (Table 2).

**Table 2:** Incidence of HIV and sexually transmitted infections among male sex workers in Mexico City

Incidence of STIs confirmed via laboratory testing	New cases (*n*)	Person-years (PY) at risk	Rate, cases/100 PY (95% *CI*)^a^
HIV	7	133.82	5.23 (2.15–10.31)
Chlamydia^b^	10	194.12	5.15 (2.58–9.34)
Gonorrhea^b^	8	203.67	3.93 (1.88–7.83)
Active Syphilis	21	161.04	13.04 (8.24–19.94)
Hepatitis B	4	189.28	2.11 (0.53–4.89)
Hepatitis C	2	209.82	0.95 (0.00–3.16)
Any STI (excluding HIV)	39	125.83	30.99 (21.73–40.26)
Any STI (including HIV)	44	87.87	50.08 (37.60–62.55)

*STI* sexually transmitted infection, *n* total respondents, *PY* person-years, *CI* confidence intervals

^a^Calculated using person-clustered, bias-corrected, accelerated bootstrapping with 1000 replications

New Cases = instances of new cases during study

^b^Site of infection is urethral, no rectal or pharyngeal testing was done for these STIs

### Associations between characteristics of MSWs and incident STIs

In the unadjusted GEE models, the odds of incident STIs did not vary significantly by older age, school education, number of clients the individual had sex with in the past week, number of non-paying partners the individual had sex with in the last week, drug use, condom use, provision of insertive anal sex, or provision of receptive anal sex (Table 3). In the multivariable adjusted model, the odds of STIs increased with age [odds ratio (*OR*) = 1.45, 95% *CI*: 1.07–1.96) and decreased with consistent condom use (*OR* = 0.03, 95% *CI*: 0.00–0.60). The associations between time-varying predictors and incident STIs are also presented as incident-rate ratios in Additional file 2, with the direction and magnitude of associations being very similar to the findings from our primary analyses presented in Table 3.

**Table 3:** Associations between socioeconomic and clinical characteristics of male sex workers and incident sexually transmitted infections

Characteristic	Unadjusted *OR* (95% *CI*)	Adjusted *OR* (95% *CI*)
Demographics		
Age, years	1.02 (0.95–1.09)	1.45 (1.07–1.96)
Highest educational attainment		
Primary or secondary school	Ref	Ref
High school	1.30 (0.62–2.70)	1.23 (0.13–11.42)
College or post-graduate	0.75 (0.29–1.94)	0.43 (0.02–9.97)
Sexual behaviors		
Had vaginal, anal, or oral sex with clients last week, number of clients	0.98 (0.87–1.10)	1.35 (0.70–2.61)
Had vaginal, anal, or oral sex with people last week, number of people	0.99 (0.94–1.03)	0.74 (0.38–1.42)
Used drugs while having sex with any of three most recent clients	0.33 (0.09–1.15)	2.07 (0.18–23.46)
Consistently used condoms during sex in past month	0.76 (0.38–1.50)	0.03 (0.00–0.68)
Had insertive anal sex with any of 3 most recent clients	0.65 (0.25–1.71)	3.26 (0.44–24.32)
Had receptive anal sex with any of 3 most recent clients	2.05 (0.75–5.64)	2.78 (0.27–28.34)
Conditional economic incentives^a^		
Control/no offer of an incentive	Ref	Ref
Offer of medium incentive for staying free of STIs	2.24 (0.80–6.30)	0.45 (0.02–11.93)
Offer of high incentive for staying free of STIs	2.89 (1.04–8.01)	0.05 (0.00–2.24)
Offer of medium incentive for study visits only	2.32 (0.84–6.45)	0.04 (0.00–1.90)

Odds ratios represent the coefficients from the generalized estimating equations (GEE) model using a logit link and binomial distribution. Odds ratios > 1 indicate an increased odds of incident STIs in the study population

Prevalent cases of HIV were retained in the analyses as still susceptible for other STIs. Prevalent cases of STI were retained in analyses as still susceptible for STIs for which they tested negative

*STI* sexually transmitted infections, *OR* odds ratio, *ref* reference level

^a^Results indicate that offering conditional economic incentives (CEIs) conditional on staying free of STIs reduces the odds of incident STIs among MSWs, when adjusting for additional demographic and behavioral risk factors. The effect of offering CEIs on incidence STIs among the study population has been described in detail by references [1, 19] listed in this article

Sensitivity analyses including incident HIV in a combined HIV/STI endpoint did not have sufficient statistical power (because about a third of the sample was HIV-positive at baseline and was therefore excluded). Our final model had the second smallest QIC criterion-indicative of model fit. The adjusted model without the covariate on number of non-paying sexual partners had a slightly smaller QIC than the final model that we used (51.363 compared to 52.461). We chose to include the variable on number of non-paying sexual partners because it is an important covariate in the literature; as such, we were balancing formal goodness-of-fit measures with epidemiological theory.

## Discussion

In this secondary analysis of a randomized controlled trial of MSWs in Mexico City, we found that incidence rates of HIV/STI were high: incidence of HIV was 5.23 cases/100 PY and incidence of syphilis was 13.04 cases/100 PY. In the adjusted multivariable regression models, the only two predictors found to be significant were age and consistent condom use. Increasing age was a risk factor for incident STIs. Conversely, consistently using condoms during anal or vaginal intercourse was protective for incident STIs.

The high HIV and active syphilis incidence rates are consistent with those of other MSWs populations in large urban areas in various places including Kenya, Cote d’Ivoire, Vietnam and the United Kingdom [4, 5, 11, 12]. Previous studies of urban, MSM populations have found a similar correlation between consistent condom use and reduced incident STIs. One study of an HIV prevention program in southern India found that an increase in consistent condom use by high risk MSM with both regular male partners (from 33 to 46%) and paying male partners (from 81 to 94%) correlated with a decline in incident syphilis cases in this population (from 14.3 to 6.8%) [27]. Another study of MSM in Australia found that, compared to men who reported consistent condom use during sex, chlamydia incidence was higher among those who reported inconsistent condom use with either regular sexual partners in the previous six months [unadjusted hazard ratio (uHR) = 1.3; 95% CI 0.9–1.8), or with casual sexual partners in the past six months (uHR = 1.6; 95% *CI*: 1.2–2.1) [28].This study also found that incident chlamydia diagnoses was higher for MSM who self-identified as sex workers [adjusted hazard ratio (aHR) = 1.6; 95% *CI*: 1.0–2.6]. The STI incidence rates from our study are also consistent with, although higher than, incidence rates found in a study among female sex workers (FSWs) in Mexico (Tijuana and Ciudad Juarez): HIV (1.12 cases/100 PY), chlamydia (9.47 cases/100 PY), active syphilis (4.01 cases/100 PY), and gonorrhea (1.78 cases/100 PY) [29].

It is important to note is that some participants were recruited into our study by referral from within *Condesa* HIV Testing Clinic and, furthermore, that participants had to agree to frequent HIV and STI testing as part of the research protocol. This means study participants likely have a greater concern and interest in their sexual health than the general MSWs population in Mexico City. As such, we expect our estimates for protective health behaviors, such as condom use, to be overestimates, and we expect estimates for risky health behaviors, such as drug use during sex work, to be underestimates when compared to the general MSWs population in Mexico City. Additionally, we expect that for some participants recruited from within *Clínica Condesa* the greater concern and interest in their sexual health, compared to the general MSWs population, stems from active STI-like symptoms.

Although our original study did not involve use of pre-exposure prophylaxis (PrEP), given the high incidence of HIV within our sample, availability of and willingness to use PrEP are likely key to shaping effective combination HIV/STI prevention strategies in the future [30]. Previous research in urban centers in Mexico indicates that awareness and willingness to initiate PrEP is high among MSM [31, 32]. Yet PrEP is currently only available in three cities in Mexico and offered to only a limited number of male sex workers and other MSM in Mexico City. Furthermore, procurement prices continue to be higher in Mexico than other Latin American countries, which can be a deterrent to larger scale PrEP implementation [31, 33]. Given the limited availability of PrEP for key populations in Mexico and the high rate of HIV observed in our sample, it is likely that MSWs in Mexico City would benefit from PrEP as part of a combination prevention strategy [34–38].

Since only a small number of MSWs reported consistently using condoms during anal and vaginal sex (28.63%), interventions that address condom use within this population are crucial for reducing STI risk. The Avahan Program—a large-scale HIV prevention program in southern India—combines peer-mediated strategies, condom distribution and STI clinical services to improve outcomes in high-risk men who have sex with men [27]. Increased condom use with commercial and non-commercial partners, as well as decreased syphilis incidence, was strongly linked with exposure to this program. In other low- and middle-income countries, evidence-based interventions for increasing condom use in sex worker populations demonstrate that reducing STI transmission is more effective when combined with the consistent and correct use of condoms [39–41]. This suggests that behavioral interventions for primary STI and HIV prevention may also serve to enhance the effectiveness of secondary prevention activities. We also recommend screening MSWs based on self-reported condom use frequency and providing a targeted HIV/STI prevention and treatment program to those who do not consistently use condoms in order to improve rates of STI testing, diagnosis, preventative education, and biomedical interventions, such as PrEP.

The primary limitation of our study was the calculation of incidence rates using data from a previous randomized controlled trial of economic incentives to reduce risky sexual practices [1]. Although the original pilot intervention was not powered to have a strong and statistically significant effect on STI/HIV acquisition compared with the control group, the intervention could have potentially resulted in fewer new STIs cases and incidence rates that underestimate the true risk in the overall population of MSWs in Mexico City. Therefore, our results should be interpreted as conservative estimates. Another limitation of the study was the small sample size, which decreased the precision of our estimates and increased the likelihood of type II error, a failure to detect a difference that was present within our sample. Some participants were lost-to-follow-up after the first and second study appointments, which further reduced the sample size. The smaller sample size, however, did allow us to collect higher quality data on STIs and potential predictors. Lastly, it is important to reiterate that it was only in our multivariable adjusted model that we found significant associations between incident STIs and predictors. Our unadjusted model did not find these associations significant.

One last element we would like to note is that our final data collection took place in May 2014. Although there have inevitably been changes to the field of HIV and in Mexico City since then, the results of this study are still relevant. There continues to be a dearth of information on STI and HIV incidence rates among MSWs in Mexico City and in Latin American more generally. To the best of our knowledge, this is the only study that provides these incidence rates for MSWs in Mexico City. Furthermore, prevention and early detection of HIV are both as important as ever. Our model is key in illuminating modifiable risk factors for prevention of STI and HIV acquisition.

## Conclusions

This study found that MSWs in Mexico City have a high incidence of STIs, particularly HIV and active syphilis. Consistently using condoms during anal and vaginal sex was found to be associated with a lower likelihood of STI acquisition among these MSWs. Consistent condom use appears to be a key potential predictor of STIs and is an important component of interventions to prevent infections. Additionally, targeted interventions for MSWs who report inconsistent condom use are warranted in light of these findings. Given such high HIV rates within this MSWs population, the population would likely benefit from future work that assesses the feasibility, effects, and cost of incorporating PrEP in multidimensional interventions.

## Supplementary Information

**Additional file 1.** Description of main covariates and outcome variables.

**Additional file 1.** Associations between socioeconomic and clinical characteristics of male sex workers and incident sexually transmitted infections.

## Data Availability

Study data is available from the authors upon request.

## Abbreviations

HIV: Human immunodeficiency virus
STI: Sexually transmitted infection
MSWs: Male sex workers
MSM: Men who have sex with men
*OR*: Odds ratio
CI: Confidence interval
INSP: Mexican National Institute of Public Health
CISIDAT: Consortium for HIV/AIDS and Tuberculosis Research
A-CASI: Audio computer assisted self-interview
RCT: Randomized controlled trial
GEE: General estimating equations
QIC: Quasi-likelihood independence model criterion
PY: Person-year
uHR: Unadjusted hazard ratio
aHR: Adjusted hazard ratio
PrEP: Pre-exposure prophylaxis
VDRL: Venereal disease research laboratory

## References

[1] Galárraga O, Sosa-Rubí SG, González A, Badial-Hernández F, Conde-Glez CJ, Juárez-Figueroa L (2014). The disproportionate burden of HIV and STIs among male sex workers in Mexico City and the rationale for economic incentives to reduce risks. J Int AIDS Soc.

[2] Bautista-Arredondo S, Colchero MA, Romero M, Conde-Glez CJ, Sosa-Rubí SG. Is the HIV epidemic stable among MSM in Mexico? HIV prevalence and risk behavior results from a nationally representative survey among men who have sex with Men. PLoS One. 2013;8:e72616. 10.1371/journal.pone.0072616 PMC3764146 24039786

[3] Baral SD, Friedman MR, Geibel S, Rebe K, Bozhinov B, Diouf D (2015). Male sex workers: practices, contexts, and vulnerabilities for HIV acquisition and transmission. Lancet.

[4] Muraguri N, Tun W, Okal J, Broz D, Raymond HF, Kellogg T (2015). HIV and STI prevalence and risk factors among male sex workers and other men who have sex with men in Nairobi, Kenya. J Acquir Immune Defic Syndr.

[5] Sethi G, Holden BM, Gaffney J, Greene L, Ghani AC, Ward H (2006). HIV, sexually transmitted infections, and risk behaviours in male sex workers in London over a 10 year period. Sex Transm Infect.

[6] Finer LB, Darroch JE, Singh S (1999). Sexual partnership patterns as a behavioral risk factor for sexually transmitted diseases. Fam Plann Perspect.

[7] Patra S (2016). Socio-cultural correlates and risky sexual behaviour influencing prevalence of HIV/AIDS and STIs in Uganda: a gender perspective. Cogent Soc Sci.

[8] Garcia PJ, Benzaken AS, Galban E, Members the A-I (2011). STI management and control in Latin America: where do we stand and where do we go from here?. Sex Transm Infect..

[9] Closson EF, Colby DJ, Nguyen T, Cohen SS, Biello K, Mimiaga MJ (2015). The balancing act: exploring stigma, economic need and disclosure among male sex workers in Ho Chi Minh City, Vietnam. Glob Public Health.

[10] WHO. Implementing comprehensive HIV/STI programmes with sex workers: practical approaches from collaborative interventions. WHO. 2013. http://www.who.int/hiv/pub/sti/sex_worker_implementation/en/. Accessed 9 May 2016.

[11] Vuylsteke B, Semde G, Sika L, Crucitti T, Ettiegne Traore V, Buve A (2012). High prevalence of HIV and sexually transmitted infections among male sex workers in Abidjan, Côte d’Ivoire: need for services tailored to their needs. Sex Transm Infect.

[12] Colby DJ, Oldenburg CE, Nguyen T, Closson EF, Biello KB, Mayer KH (2016). HIV, hepatitis c, and other sexually transmitted infections among male sex workers in Ho Chi Minh City, Vietnam. AIDS Behav.

[13] Monteiro JFG, Marshall BDL, Escudero D, Sosa-Rubí SG, González A, Flanigan T (2015). Preventing HIV transmission among partners of HIV-positive male sex workers in Mexico City: a modeling study. AIDS Behav.

[14] Verma RK, Collumbien M (2004). Homosexual activity among rural Indian men: implications for HIV interventions. AIDS.

[15] Setia MS, Sivasubramanian M, Anand V, Row-Kavi A, Jerajani HR (2010). Married men who have sex with men: the bridge to HIV prevention in Mumbai, India. Int J Public Health.

[16] Hemmige V, Snyder H, Liao C, Mayer K, Lakshmi V, Gandham SR (2011). Sex position, marital status, and HIV risk among Indian men who have sex with men: clues to optimizing prevention approaches. AIDS Patient Care STDS.

[17] van Dam CJ, Holmes KK (2000). STD prevention: effectively reaching the core and a bridge population with a four-component intervention. Sex Transm Dis.

[18] Sexually Transmitted Infections in Developing Countries: current concepts and strategies on improving STI prevention, treatment, and control. CDC, World Bank. p. 55.

[19] Galárraga O, Sosa-Rubí SG, Infante C, Gertler PJ, Bertozzi SM (2014). Willingness-to-accept reductions in HIV risks: conditional economic incentives in Mexico. Eur J Health Econ.

[20] Infante C, Sosa-Rubi SG, Cuadra SM (2009). Sex work in Mexico: vulnerability of male, travesti, transgender and transsexual sex workers. Cult Health Sex.

[21] Bazzi AR, Rangel G, Martinez G, Ulibarri MD, Syvertsen JL, Bazzi SA (2015). Incidence and predictors of HIV and sexually transmitted infections among female sex workers and their intimate male partners in Northern Mexico: a longitudinal, multilevel study. Am J Epidemiol.

[22] Efron B, Tibshirani R (1986). Bootstrap methods for standard errors, confidence intervals, and other measures of statistical accuracy. Stat Sci.

[23] Davison AC, Hinkley DV (1997). Bootstrap methods and their application.

[24] Galárraga O, Sosa-Rubí SG, Kuo C, Gozalo P, González A, Saavedra B (2017). *Punto Seguro*: a randomized controlled pilot using conditional economic incentives to reduce sexually transmitted infection risks in Mexico. AIDS Behav.

[25] Liang K-Y, Zeger SL (1986). Longitudinal data analysis using generalized linear models. Biometrika.

[26] Cui J (2007). QIC program and model selection in GEE analyses. Stata J.

[27] Subramanian T, Ramakrishnan L, Aridoss S, Goswami P, Kanguswami B, Shajan M (2013). Increasing condom use and declining STI prevalence in high-risk MSM and TGs: evaluation of a large-scale prevention program in Tamil Nadu, India. BMC Public Health.

[28] Wilkinson A, El-Hayek C, Fairley CK, Leslie D, Roth N, Tee BK (2012). Incidence and risk factors associated with chlamydia in men who have sex with men: a cohort analysis of Victorian Primary Care Network for Sentinel Surveillance data. Sex Transm Infect.

[29] Strathdee SA, Abramovitz D, Lozada R, Martinez G, Rangel MG, Vera A (2013). Reductions in HIV/STI incidence and sharing of injection equipment among female sex workers who inject drugs: results from a randomized controlled trial. PLoS ONE.

[30] Edeza A, Galárraga O, Santamaria EK, Sosa-Rubí S, Operario D, Biello KB (2020). “I do try to use condoms, but…”: Knowledge and interest in PrEP among male sex workers in Mexico City. Arch Sex Behav.

[31] Ravasi G, Grinsztejn B, Baruch R, Guanira JV, Luque R, Cáceres CF (2016). Towards a fair consideration of PrEP as part of combination HIV prevention in Latin America. J Int AIDS Soc.

[32] Pitpitan EV, Goodman-Meza D, Burgos JL, Abramovitz D, Chavarin CV, Torres K (2015). Prevalence and correlates of HIV among men who have sex with men in Tijuana, Mexico. J Int AIDS Soc.

[33] Pan American Health Organization, World Health Organization. Antiretroviral treatment in the spotlight: a public health analysis in Latin America and the Caribbean, 2013. 2013. http://iris.paho.org/xmlui/handle/123456789/31367?locale-attribute=pt. Accessed 3 May 2019.

[34] Hankins C, Macklin R, Warren M (2015). Translating PrEP effectiveness into public health impact: key considerations for decision-makers on cost-effectiveness, price, regulatory issues, distributive justice and advocacy for access. J Int AIDS Soc.

[35] Galea JT, Kinsler JJ, Salazar X, Lee S-J, Giron M, Sayles JN (2011). Acceptability of pre-exposure prophylaxis as an HIV prevention strategy: barriers and facilitators to pre-exposure prophylaxis uptake among at-risk peruvian populations. Int J STD AIDS.

[36] Liu AY, Kittredge PV, Vittinghoff E, Raymond HF, Ahrens K, Matheson T (2008). Limited knowledge and use of HIV post- and pre-exposure prophylaxis among gay and bisexual men. J Acquir Immune Defic Syndr.

[37] Hankins CA, de Zalduondo BO (2010). Combination prevention: a deeper understanding of effective HIV prevention. AIDS.

[38] Fast-tracking combination prevention: towards reducing new HIV infections to fewer than 500 000 by 2020. UNAIDS. 2015. http://www.unaids.org/en/resources/documents/2015/20151019_JC2766_Fast_tracking_combination_prevention. Accessed 9 May 2016.

[39] Wariki WM, Ota E, Mori R, Koyanagi A, Hori N, Shibuya K. Behavioral interventions to reduce the transmission of HIV infection among sex workers and their clients in low- and middle-income countries. Cochrane Database Syst Rev. 2012;(2):CD005272. 10.1002/14651858.CD005272.pub3 PMC11345029 22336811

[40] Ghys PD, Diallo MO, Ettiègne-Traoré V, Satten GA, Anoma CK, Maurice C (2001). Effect of interventions to control sexually transmitted disease on the incidence of HIV infection in female sex workers. AIDS.

[41] Laga M, Alary M, Behets F, Goeman J, Piot P, Nzila N (1994). Condom promotion, sexually transmitted diseases treatment, and declining incidence of HIV-1 infection in female Zairian sex workers. Lancet.

