# Supplementary material for: Incidence and time-varying predictors of HIV and sexually transmitted infections among male sex workers in Mexico City
Source: Infect Dis Poverty. 2021 Jan 19;10:7. doi: 10.1186/s40249-020-00792-2 (PMC7814587; doi:10.1186/s40249-020-00792-2)
Supplement: Supplementary file 1 — Additional file 1. Description of main covariates and outcome variables. [file 40249_2020_792_MOESM1_ESM.pdf]

Additional file 1. Description of main covariates and outcome variables

Variable	Question	Answer options	Coding for the analysis
Demographics			
Age	How old are you?	Discrete variable; 99.I don't want to answer	Discrete variable
Highest educational attainment	What is the highest level of education you have completed?	0.Didn't complete primary; 1.Primary; 2.Secondary; 3.High School; 4.College; 5.Graduate School; 99.I don't wish to answer	1.Primary or secondary school; 2.High school; 3.College or higher
Sexual behaviors			
Had vaginal, anal, or oral sex with clients last week	With how many clients did you have vaginal or anal or oral sex with during the last week?	Number of male clients (discrete variable); Number of female clients (discrete variable); 888.I don't know; 999. I don't wish to answer	Total number of clients (discrete variable)
Had vaginal, anal, or oral sex with people last week	With how many non-paying partners did you have vaginal or anal or oral sex with during the last week?	Number of male non-paying partners (discrete variable); Number of female non-paying partners (discrete variable); 888.I don't know; 999. I don't wish to answer	Total number of non-paying partners (discrete variable)
Used drugs while having sex with any of 3 most recent clients	Before being with your last client, did you take any drugs?†	1.Yes; 3.No; 99.I don't wish to answer	0.No; 1.Yes (used drugs before having sex with at least one of 3 most recent clients)
Consistently used condoms during sex in past month	When you had sex, how often did you use condoms in the past month?	0.Never; 1.Almost never; 2.Sometimes; 3.Almost every time; 4.Every time	0.Almost every time, sometimes, almost never, or never; 1.Every time
Had insertive anal sex with any of 3 most recent clients	Did you provide the services that your most recent client requested? If yes, how many times?†	Discrete variables: 1.Talk, 2.Insertive anal sex (he penetrated you), 3.Receptive anal sex (you penetrated him), 4.Oral sex, 5.Masturbating, 6.Dance, 7.Bathing, 8.Massage, 9.Striptease, 10.Vaginal sex. 99.Do not wish to answer	0.No; 1.Yes (had insertive anal sex with at least one of 3 most recent clients)

Additional file 1. (Continued)

Variable	Question	Answer Options	Coding for the Analysis
Sexual behaviors (continued)			
Had receptive anal sex with any of 3 most recent clients	Did you provide the services that your most recent client requested? If yes, how many times?[†]	Discrete variables: 1.Talk, 2.Insertive anal sex (he penetrated you), 3.Receptive anal sex (you penetrated him), 4.Oral sex, 5.Masturbating, 6.Dance, 7.Bathing, 8.Massage, 9.Striptease, 10.Vaginal sex. 99.Do not wish to answer	0.No; 1.Yes (had receptive anal sex with at least one of 3 most recent clients)
Sexually transmitted infections (STI) incidence			
New STIs and HIV	Participants provided blood and urine samples, collected following the local biosafety protocols by trained staff and analyzed by lab technicians. Urine specimens were tested for gonorrhea and chlamydia (PCR Cobas-Amplicor; Roche, Basel, Switzerland); blood specimens served to measure presence of HIV, hepatitis B, hepatitis C and syphilis antibodies (Abbott HIV-1 and HIV-2, Ag/Ab Combo, anti-HBc, anti-HCV and syphilis TP quimioluminiscence immunoassay; Abbott Laboratories, North Chicago, IL, USA) running in Architect i2000 (Abbott). HIV+ samples were confirmed with HIV-1 and HIV-2 CombFirm (Orgenics, Yavne, Israel); and anti-HBcwas tested with Determine HBsAg and syphilis TP (Abbott) with tittered VDRL, the Venereal Disease Research Laboratory test. Two subgroups were defined for the markers of Syphilis: antibody positivity was regarded as a lifetime marker of past or present infection, whereas treponemic antibody positivity together with VDRL demonstrated active syphilis.		0.No 1.Yes (any new STI/HIV)
Randomization			
Conditional Economic Incentives	Participants were randomized and allocated into one of four groups: control, medium incentive to stay free of new curable STIs (USD $50), high incentive to stay free of new curable STIs (USD $75), or medium incentive to attend study visits (USD $50).[§]		1.Control/no incentive; 2.Medium incentive to stay free of new curable STIs; 3.High incentive to stay free of new curable STIs; 4.Medium incentive to attend study visits

Notes: [†]The same question was asked about next-to-last client and second-to-last client; [§]Approximate average exchange rate at time of study (2012–2014): 12 Mexican Pesos per $1 (USD).
