# Supplementary material for: Incidence and time-varying predictors of HIV and sexually transmitted infections among male sex workers in Mexico City
Source: Infect Dis Poverty. 2021 Jan 19;10:7. doi: 10.1186/s40249-020-00792-2 (PMC7814587; doi:10.1186/s40249-020-00792-2)
Supplement: Supplementary file 2 — Additional file 1. Associations between socioeconomic and clinical characteristics of male sex workers and incident sexually transmitted infections. [file 40249_2020_792_MOESM2_ESM.pdf]

Additional file 2. Associations between socioeconomic and clinical characteristics of male sex workers and incident sexually transmitted infections *

Characteristic	Unadjusted IRR (95% *CI*)	Adjusted IRR (95% *CI*)
Demographics		
Age, years	1.01 (0.9, 1.08)	1.28 (1.03, 1.58)
Highest educational attainment		
Primary or secondary school	ref	ref
High school	1.24 (0.64–2.44)	1.15 (0.19–7.04)
College or post-graduate	0.78 (0.32–1.90)	0.61 (0.05–7.90)
Sexual behaviors		
Had vaginal, anal, or oral sex with clients last week, number of clients	0.98 (0.88–1.09)	1.18 (0.68–2.05)
Had vaginal, anal, or oral sex with people last week, number of people	0.99 (0.95–1.03)	0.84 (0.49–1.44)
Used drugs while having sex with any of three most recent clients	0.36 (0.11–1.22)	1.48 (0.18–12.18)
Consistently used condoms during sex in past month	0.79 (0.42–1.48)	0.11 (0.01–0.96)
Had insertive anal sex with any of 3 most recent clients	0.69 (0.28–1.70)	1.93 (0.40–9.24)
Had receptive anal sex with any of 3 most recent clients	1.86 (0.72–4.81)	1.76 (0.33–9.46)
Conditional economic incentives*		
Control/No offer of an incentive	ref	ref
Offer of medium incentive for staying free of STIs	2.05 (0.77–5.46)	0.52 (0.03–9.28)
Offer of high incentive for staying free of STIs	2.54 (0.97–6.62)	0.13 (0.01–3.16)
Offer of medium incentive for study visits only	2.11 (0.80–5.56)	0.13 (0.01–2.54)

Abbreviations: STI, sexually transmitted infections; IRR, incidence-rate ratio; ref, reference level.
Incidence-rate ratios represent the coefficients from the generalized estimating equations (GEE) model using a log link and Poisson distribution. Incidence-rate ratios > 1 indicate an increased risk of incident STIs.
*Prevalent cases of HIV were retained in the analyses as still susceptible for other STIs. Prevalent cases of STI were retained in analyses as still susceptible for STIs for which they tested negative.
**Results indicate that offering conditional economic incentives (CEIs) conditional on staying free of STIs reduces the risk of incident STIs among MSWs, when adjusting for additional demographic and behavioral risk factors. The effect of offering CEIs on incidence STIs among the study population has been described in detail by references [1] and [19] listed in this article.
